# Relative molecule self-attention transformer

**DOI:** 10.1186/s13321-023-00789-7

**Published:** 2024-01-03

**Authors:** Łukasz Maziarka, Dawid Majchrowski, Tomasz Danel, Piotr Gaiński, Jacek Tabor, Igor Podolak, Paweł Morkisz, Stanisław Jastrzębski

**Affiliations:** 1 Faculty of Mathematics and Computer Science, Jagiellonian University, Łojasiewicza 6, 30-348 Cracow, Poland; 2 NVIDIA, 2788 San Tomas Expy, Santa Clara, CA 95051 USA; 3 Ardigen, Podole 76, 30-394 Cracow, Poland; 4 Molecule.one, Al. Jerozolimskie 96, 00-807 Warsaw, Poland

**Keywords:** Molecular property prediction, Molecular self-attention, Neural networks pre-training

## Abstract

The prediction of molecular properties is a crucial aspect in drug discovery that can save a lot of money and time during the drug design process. The use of machine learning methods to predict molecular properties has become increasingly popular in recent years. Despite advancements in the field, several challenges remain that need to be addressed, like finding an optimal pre-training procedure to improve performance on small datasets, which are common in drug discovery. In our paper, we tackle these problems by introducing Relative Molecule Self-Attention Transformer for molecular representation learning. It is a novel architecture that uses relative self-attention and 3D molecular representation to capture the interactions between atoms and bonds that enrich the backbone model with domain-specific inductive biases. Furthermore, our two-step pretraining procedure allows us to tune only a few hyperparameter values to achieve good performance comparable with state-of-the-art models on a wide selection of downstream tasks.

**Supplementary Information:**

The online version contains supplementary material available at 10.1186/s13321-023-00789-7.

## Scientific contribution

A novel graph transformer architecture for molecular property prediction is introduced. The task-agnostic methodology for pre-training this model is presented, improving target task performance with minimal hyperparameter tuning. A rigorous exploration of the design space for the self-attention layer is conducted to identify the optimal architecture.

**Supplementary Information:**

The online version contains supplementary material available at 10.1186/s13321-023-00789-7.

## Introduction

Predicting molecular properties is central to applications such as drug discovery or material design. Without accurate prediction of properties such as toxicity, a promising drug candidate is likely to fail clinical trials. Many molecular properties cannot be feasibly computed (simulated) from first principles as their complexity scales with at least the 4th power of the number of atoms. It makes computation infeasible for even moderately large systems. Moreover, complex molecular properties, such as predicting the yield of chemical reactions, are still beyond the reach of what is typically referred to as computational chemistry methods [1]. Instead, these properties have to be extrapolated from an often small experimental dataset [2, 3]. The prevailing approach is to train a machine learning model such a random forest [4] or a graph neural network [5] from scratch to predict the desired property for a new molecule [6].

Machine learning is moving away from training models purely from scratch. In natural language processing (NLP), advances in large-scale pretraining [7, 8] and the development of the Transformer [9] architecture have culminated in large gains in data efficiency across multiple tasks because pretrained models usually need less data to produce similar results as models trained from scratch [10]. Instead of training models purely from scratch, the models in NLP are commonly first pretrained on large unsupervised corpora. The chemistry domain might be on the brink of an analogous revolution, which could be especially transformative due to the high cost of obtaining large experimental datasets. In particular, recent work has proposed Molecule Attention Transformer (MAT), a Transformer-based architecture adapted to processing molecular data [11, 12] and pretrained using self-supervised learning for graphs [13]. Several works have shown further gains by improving network architecture or the pretraining tasks [14–16].

However, pretraining has not yet led to such transformative data-efficiency gains in molecular property prediction. For instance, non-pretrained models with extensive handcrafted featurization tend to achieve very competitive results [17]. We reason that architecture might be a key bottleneck. In particular, most Transformers for molecules do not encode the three-dimensional structure of the molecule [14, 16], which is a key factor determining many molecular properties. On the other hand, performance has been significantly boosted in other domains by enriching the Transformer architecture with proper inductive biases [18–27]. Motivated by this perspective, we methodologically explore the design space of the self-attention layer, a key computational primitive of the Transformer architecture, for molecular property prediction. In particular, we explore variants of relative self-attention, which has been shown to be effective in various domains such as protein design and NLP [19, 21].

**Fig. 1:**
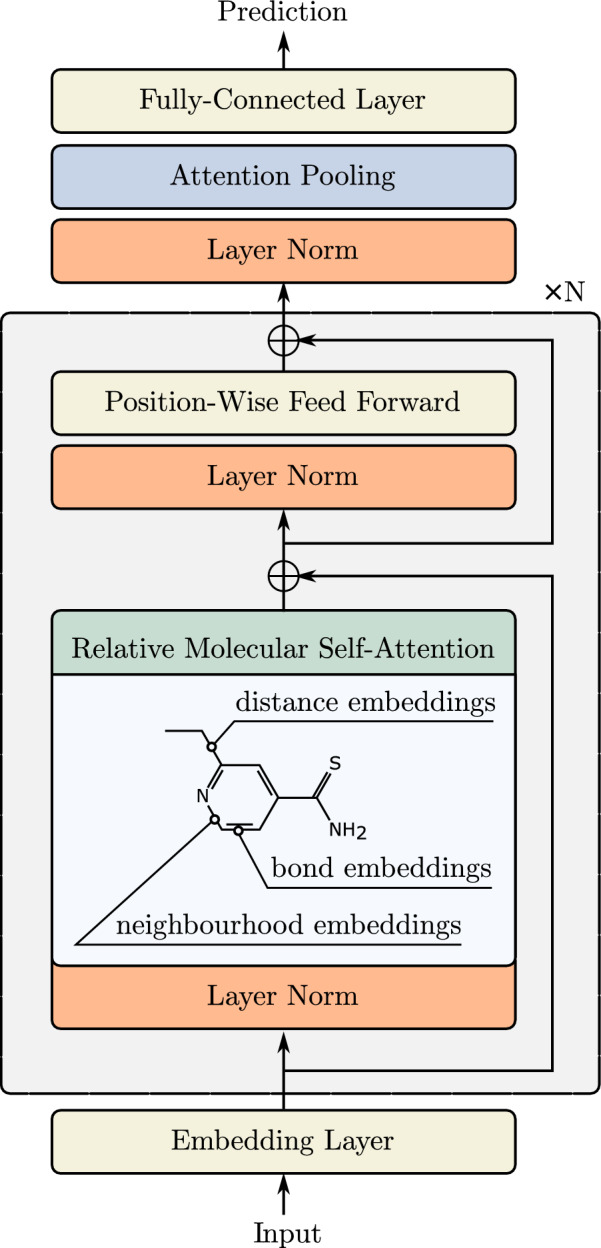
Relative Molecule Self-Attention Transformer uses a novel relative self-attention block tailored to molecule property prediction. It fuses three types of features: distance embedding, bond embedding, and neighborhood embedding

**Fig. 2:**
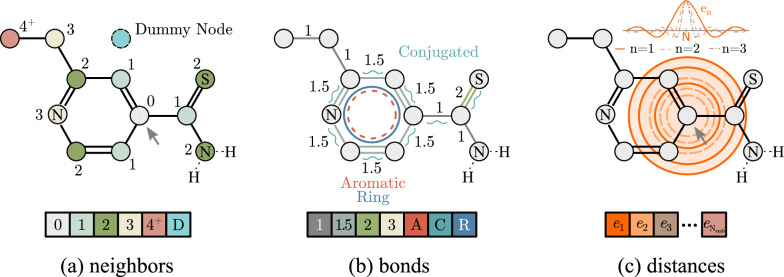
The Relative Molecule Self-Attention layer is based on the following features: **a** neighborhood embedding one-hot encodes graph distances (neighborhood order) from the source node marked with an arrow; **b** bond embedding one-hot encodes the bond order (numbers next to the graph edges) and other bond features for neighboring nodes; **c** distance embedding uses radial basis functions to encode pairwise distances in the 3D space. These features are fused according to Eq. (5)

Our main contribution is a new self-attention layer for molecular graphs. We tackle the aforementioned issues with Relative Molecule Self-Attention Transformer (R-MAT), our pre-trained transformer-based model, shown in Fig. 1. We propose Relative Molecule Self-Attention, a novel variant of relative self-attention, which allows us to effectively fuse distance and graph neighborhood information (see Fig. 2). We perform pretraining using local atom context masking and global graph-based prediction, which results in one strong architecture for which we only tune a range of learning rate values. Our model achieves competitive performance across a wide range of tasks. Satisfyingly, R-MAT outperforms more specialized models without using extensive handcrafted featurization or adapting the architecture specifically to perform well on quantum prediction benchmarks. The importance of effectively representing distance and other relationships in the attention layer is evidenced by large performance gains compared to MAT.

## Methods

### Background

#### Transformers

The Transformer architecture was introduced by Vaswani et al. [9] and has since become the standard architecture for NLP tasks. The model uses a self-attention mechanism to process the input sequence, allowing it to capture long-term dependencies without the need for recurrent layers. This has resulted in improved performance and faster training times compared to traditional NLP models. Originally it was trained for machine translation tasks. However since its inception, numerous successors of the Transformer model have been developed, such as BERT [7] or GPT [28], which showed that a properly pretrained Transformer can obtain state-of-the-art on a wide selection of NLP tasks.

Pretraining coupled with the efficient Transformer architecture [9] unlocked state-of-the-art performance also in molecular property prediction [12, 14–16, 29, 30]. First applications of deep learning did not offer large improvements over more standard methods such as random forests [31–33]. Consistent improvements were in particular enabled by more efficient architectures adapted to this domain [17, 34, 35]. In this spirit, our goal is to further advance modeling for any chemical task by redesigning self-attention for molecular data.

Encoding efficiently the relation between tokens in self-attention has been shown to substantially boost the performance of Transformers in vision, language, music, and biology [19–25]. The vanilla self-attention includes absolute encoding of position, which can hinder learning when the absolute position in the sentence is not informative.^1^ Relative positional encoding featurizes the relative distance between each pair of tokens, which led to substantial gains in the language and music domains [22, 36].

On the other hand, a Transformer can be perceived as a fully-connected (all vertices are connected to all vertices) Graph Neural Network with trainable edge weights given by a self-attention [37]. From a practical perspective, the empirical success of the Transformer stems from its ability to learn highly complex and useful patterns.

#### Molecular self-attention

In this section, we give a short background on the prior works on adapting self-attention for molecular data and point out their potential shortcomings.

**Text Transformers.** Multiple works have applied the Transformer directly to molecules encoded as text using the SMILES representation [14, 15, 29, 30, 38]. SMILES is a linear encoding of a molecule into a string of characters according to a deterministic ordering algorithm [39, 40]. For example, the SMILES encoding of carbon dioxide is C(=O)=O.

Adding a single atom can completely change the ordering of atoms in the SMILES encoding. Hence, the relative positions of individual characters are not easily related to their proximity in the graph or space. This is in contrast to natural language processing, where the distance between two words in the sentence can be highly informative [19, 22, 25]. We suspect this makes the use of self-attention in SMILES models less effective. Another readily visible shortcoming is that the graph structure and distances between molecule atoms are either completely encoded or thrown out.

**Graph Transformers.** Several works have proposed Transformers that operate directly on a graph [12, 16, 41]. The GROVER and the U2GNN models take as input a molecule encoded as a graph [16, 41]. In both of them, the self-attention layer does not have a direct access to the information about the graph. Instead, the information about the relations between atoms (existence of a bond or distance in the graph) is indirectly encoded by a graph convolutional layer that is run in GROVER within each layer, and in U2GNN only at the beginning. Similarly to Text Transformers, Graph Transformers also do not take into account the distances between atoms.

Structured Transformer introduced by Ingraham et al. [21] uses relative self-attention that operates on amino acids in the task of protein design, while we focus on classifiers in the context of molecular property prediction. Its self-attention, similarly to our work, provides the model with information about the three-dimensional structure of the molecule. As R-MAT encodes the relative distances between pairs of atoms, Structured Transformer also uses relative distances between modeled amino acids and their position in the sequence. However, it encodes them in a slightly different way. We incorporate their ideas and extend them to enable the processing of molecular data.

**Molecule Attention Transformer.** Our work is closely related to Molecule Attention Transformer (MAT), a transformer-based model with self-attention tailored to processing molecular data [12]. In contrast to most of the aforementioned models, MAT incorporates distance information in its self-attention module. MAT stacks *N* Molecule Self-Attention blocks followed by a mean pooling and a prediction layer.

For a *D*-dimensional sequence embedding ${\textbf{X}} \in {\mathbb {R}}^{N\times D}$, the standard self-attention operation is defined as

1 $$\begin{aligned} {\mathcal {A}}({\textbf{X}}) = {\text {Softmax}}\left( \frac{{\textbf{Q}}{\textbf{K}}^T}{\sqrt{d_k}} \right) {\textbf{V}}, \end{aligned}$$

where ${\textbf{Q}}={\textbf{X}}{\textbf{W}}^Q$, ${\textbf{K}}={\textbf{X}}{\textbf{W}}^K$, and ${\textbf{V}}={\textbf{X}}{\textbf{W}}^V$. Molecule Self-Attention extends Eq. (1) to include additional information about bonds and distances between atoms in the molecule as

2 $$\begin{aligned} {\mathcal {A}}({\textbf{X}}) = \left( \lambda _{a} {\text {Softmax}} \left( \frac{{\textbf{Q}}{\textbf{K}}^T}{\sqrt{d_k}}\right) + \lambda _d\, g({\textbf{D}}) + \lambda _g {\textbf{A}} \right) {\textbf{V}}, \end{aligned}$$

where $\lambda _a$, $\lambda _d$, $\lambda _g$ are the weights given to individual parts of the attention module, *g* is a function given by either a softmax, or an element-wise $g(d) = \exp (-d)$, ${\textbf{A}}$ is the adjacency matrix (with ${\textbf{A}}_{(i,j)} = 1$ if there exists a bond between atoms *i* and *j* and 0 otherwise) and ${\textbf{D}}$ is the distance matrix, where ${\textbf{D}}_{(i,j)}$ represents the distance between the atoms *i* and *j* in the 3D space. Ultimately, Molecule Attention Transformer incorporates the interatomic distances and atom adjacency by calculating the weighted average of the classical self-attention, a function of atoms’ distance, and a function of atoms’ neighborhood in its Molecule Self-Attention layer.

Self-attention can relate input elements in a highly flexible manner. In contrast, there is little flexibility in how Molecule Self-Attention can use the information about the distance between two atoms. The strength of the attention between two atoms depends monotonically on their relative distance. However, molecular properties can depend in a highly nonlinear way on the distance between atoms. This has motivated works such as Klicpera et al. [35] to explicitly model the interactions between atoms, using higher-order terms.

#### Relative positional encoding

In natural language processing, a vanilla self-attention layer does not take into account the positional information of the input tokens (i.e. if we permute the layer input, the output will stay the same). In order to add the positional information into the input data, the vanilla transformer encodes the absolute position of the input tokens and adds its embeddings into the input token embeddings before passing this data into the self-attention layers. On the other hand, self-attention with relative positional encoding [19] adds the embedding of the relative distance between each pair of tokens directly into the self-attention layer, which leads to substantial gains in the learned task. In our work, we use relative self-attention to encode the information about the relative neighborhood, distances, and physicochemical features between all pairs of atoms in the input molecule (See Fig. 2).

#### Successors

Since the initial version of this paper was made public, several researchers have adopted their own versions of molecular self-attention to solve molecular property prediction tasks [42, 43], for some datasets even surpassing the results of our model. Choukroun et al. [42] proposed a model with a different self-attention mechanism, more similar to Maziarka et al. [12], that, trained with their custom data augmentation, outperforms R-MAT in the QM9 task. Wu et al. [43] proposed Molformer – an architecture that exploits both molecular 3D geometry and its motifs. Their model surpasses R-MAT in the QM7, BBBP, and BACE tasks.

### Atom relation embedding

Our core idea to improve Molecule Self-Attention is to add flexibility in how it processes graph and distance information. Specifically, we adapt positional relative encoding to processing molecules [19, 20, 22, 25], which we note was already hinted at by Shaw et al. [19] as a high-level future direction. The key idea in these works is to enrich the self-attention block to efficiently represent information about the relative positions of items in the input sequence.

What reflects the relative position of two atoms in a molecule? Similarly to MAT, we delineate three interrelated factors: (1) their relative distance, (2) their distance in the molecular graph, and (3) their physiochemical relationship (e.g. whether they are within the same aromatic ring). We will also enrich our self-attention with this information. However, instead of modeling it as a weighted average, like in Molecule Attention Transformer, we allow the network to learn how to use this information by itself.

In the next step, we depart from Molecule Self-Attention [12] and introduce new factors to the relation embedding. Given two atoms, represented by vectors ${\varvec{x}}_i, {\varvec{x}}_j \in {\mathbb {R}}^D$, we encode their relation using an *atom relation embedding* ${\varvec{b}}_{ij} \in {\mathbb {R}}^{D'}$. This embedding will then be used in the relative self-attention module after a projection layer.

In the next step, we describe three components that are concatenated to form the embedding ${\varvec{b}}_{ij}$.

**Table 1:** Featurization used to embed neighborhood order in R-MAT

Indices	Description
0	$i = j$
1	Atoms *i* and *j* are connected with a bond
2	In the shortest path between atoms *i* and *j* there is one atom
3	In the shortest path between atoms *i* and *j* there are two atoms
4	In the shortest path between atoms *i* and *j* there are three or more atoms
5	Any of the atoms *i* or *j* is a dummy node

**Neighborhood embeddings.** First, we encode the neighborhood order between two atoms as a 6-dimensional one hot encoding, with information about how many other vertices are between nodes *i* and *j* in the original molecular graph (see Fig. 2). The list of neighborhood features is presented in Table 1.

**Bond embeddings.** Finally, we featurize each bond to reflect the physical relation between pairs of atoms that might arise from, for example, being part of the same aromatic structure in the molecule. Molecular bonds are embedded in as a 7-dimensional vector following Coley et al. [44], described in Table 2. When the two atoms are not connected by a true molecular bond, all 7 dimensions are set to zeros. We note that while these features can be easily learned in pretraining, we hypothesize that this featurization might be highly useful for training R-MAT on smaller datasets.

**Table 2:** Featurization used to embed molecular bonds in R-MAT

Indices	Description
$0 - 3$	Bond order as one-hot vector of 1, 1.5, 2, 3
4	Is aromatic
5	Is conjugated
6	Is in a ring

**Distance embeddings.** As we discussed earlier, we hypothesize that a much more flexible representation of the distance information should be facilitated in MAT. To achieve this, we use a radial basis distance encoding proposed by Klicpera et al. [35]:

$$\begin{aligned} e_{n}(d) = \sqrt{\frac{2}{c}} \cdot \frac{\sin {(\frac{n \pi }{c} d)}}{d}, \end{aligned}$$

where *d* is the 3D Euclidean distance between two atoms, *c* is the predefined cutoff distance, $n \in \{1, \ldots , \text {N}_{\text {emb}}\}$ and $\hbox {N}_{\text {emb}}$ is the total number of radial basis functions that we use. To improve the differentiability, the obtained numbers are multiplied by the polynomial envelope function

$$\begin{aligned} u(d) =\, 1 - \frac{(p+1)(p+2)}{2} \left( \frac{d}{c}\right) ^p + p(p+2) \left( \frac{d}{c}\right) ^{p+1} - \frac{p(p+1)}{2} \left( \frac{d}{c}\right) ^{p+2}, \end{aligned}$$

with $p=6$, resulting in the final distance embedding.

This results in the distance embedding given by a whole vector (with $\hbox {N}_{\text {emb}}$ dimensions), instead of just one number, like in the case of Molecule Attention Transformer.

### Relative molecule self-attention

Equipped with the embedding ${\varvec{b}}_{ij}$, which is a concatenation of neighborhood, distance, and bond embeddings, for each pair of atoms in the molecule, we now use it to define a novel self-attention layer that we refer to as Relative Molecule Self-Attention.

First, mirroring the key-query-value design in the vanilla self-attention (c.f. Eq. (1)), we transform ${\varvec{b}}_{ij}$ into a key and value specific vectors ${\varvec{b}}^V_{ij}, {\varvec{b}}^K_{ij}$ using two neural networks $\phi _V$ and $\phi _K$. Each neural network consists of two layers. A hidden layer, shared between all attention heads and the output layer, that create a separate relative embedding for different attention heads.

**Fig. 3:**
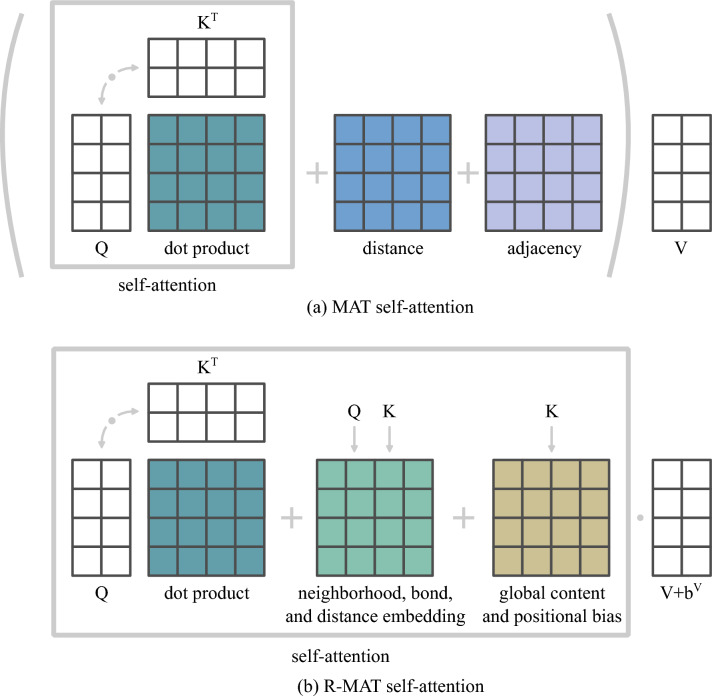
Comparison between MAT and R-MAT self-attention modules. The self-attention block comprises scaling and applying softmax. In MAT, distance and adjacency matrices are outside the self-attention block, while in R-MAT all matrices are mixed within the self-attention. Moreover, all atom-pair embeddings are collected in one matrix that is also multiplied by queries and keys

Consider Eq. (1) in index notation:

3 $$\begin{aligned} {\mathcal {A}}({\textbf{X}})_i = \sum _{j=1}^{n} {\text {Softmax}}\left( \frac{e_{ij}}{\sqrt{d_z}}\right) ^T (x_j W^V), \end{aligned}$$

where the unnormalized attention is $e_{ij} = (x_i W^Q)(x_j W^K)^T$. By analogy, in Relative Molecule Self-Attention, we compute $e_{ij}$ as

4 $$\begin{aligned} e_{ij} = \underbrace{(x_i W^Q)(x_j W^K)^T}_{\text {vanilla self-attention}} + \underbrace{(x_i W^Q) {\varvec{b}}_{ij}^K}_{\begin{array}{c} \text {content-dependent} \\ \text {positional bias} \\ \text {for query} \end{array}} + \underbrace{(x_j W^K) {\varvec{b}}_{ij}^K}_{\begin{array}{c} \text {content-dependent} \\ \text {positional bias} \\ \text {for key} \end{array}} + \underbrace{{\varvec{u}}^T(x_j W^K)}_{\begin{array}{c} \text {global content} \\ \text {bias} \end{array}} + \underbrace{{\varvec{v}}^T {\varvec{b}}_{ij}^K}_{\begin{array}{c} \text {global positional} \\ \text {bias} \end{array}}, \end{aligned}$$

where ${\varvec{u}}, {\varvec{v}} \in {\mathbb {R}}^{D'}$ are trainable vectors. We then define Relative Molecule Self-Attention operation:

5 $$\begin{aligned} {\mathcal {A}}({\textbf{X}})_i = \sum _{j=1}^{n} \textrm{Softmax}\left( \frac{e_{ij}}{\sqrt{d_z}}\right) ^T (x_j W^V + {\varvec{b}}_{ij}^V). \end{aligned}$$

In other words, we enrich the self-attention layer with atom relations embedding. Inspired by the text transformer advancements, we add content-dependent positional bias, global content bias, and global positional bias [20, 22] (that are calculated based on ${\varvec{b}}_{ij}^K$) to the layer in the phase of attention weights calculation. Then, during calculation of the attention weighted average, we also include the information about the other embedding ${\varvec{b}}_{ij}^V$. This variant of relative self-attention allows us to model the interaction of query, key, and relative position embeddings simultaneously, which was not possible with the original relative self-attention proposed by Shaw et al. [19]. The self-attention modules of MAT and R-MAT are compared in Fig. 3.

### Relative molecule self-attention transformer

Finally, we use Relative Molecule Self-Attention to construct Relative Molecule Self-Attention Transformer (R-MAT). The key changes compared to MAT are: (1) the use of Relative Molecule Self-Attention, (2) extended atom featurization, and (3) extended pretraining procedure. Figure 1 illustrates the R-MAT architecture.

The input is embedded as a matrix of size $N_{\text {atom}} \times 36$ where each atom of the input is embedded following Coley et al. [45] and Pocha et al. [45] (see the details in Additional file 1). We process the input using *N* stacked Relative Molecule Self-Attention attention layers. Each attention layer is followed by a position-wise feed-forward Network (similar as in the classical transformer model [9]), which consists of 2 linear layers with a leaky-ReLU nonlinearity between them.

After processing the input using attention layers, we pool the representation into a constant-sized vector. We replace simple mean pooling with an attention-based pooling layer. After applying *N* self-attention layers, we use the following self-attention pooling [46] in order to get the graph-level embedding of the molecule:

$$\begin{aligned} {\textbf{P}}= & {} {\text {Softmax}}(W_2 \tanh (W_1 {\textbf{H}}^T )), \\ {\textbf{g}}= & {} {\text {Flatten}}({\textbf{P}}{\textbf{H}}), \end{aligned}$$

where ${\textbf{H}}$ is the hidden state obtained from self-attention layers, $W_1 \in {\mathbb {R}}^{P \times D}$ and $W_2 \in {\mathbb {R}}^{S \times P}$ are pooling attention weights, with *P* equal to the pooling hidden dimension and *S* equal to the number of pooling attention heads. Finally, the graph embedding ${\textbf{g}}$ is then passed to the two-layer MLP, with leaky-ReLU activation, in order to make the prediction.

**Pretraining.** We used a two-step pretraining procedure. In the first step, our network is trained with the contextual property prediction task proposed by Rong et al. [16], where we mask not only selected atoms but also their neighbors. The goal of the task is to predict the whole atom context. This task is much more demanding for the network than the classical masking approach presented by Maziarka et al. [12] since the network has to encode more specific information about the masked atom neighborhood. Furthermore, the size of the context vocabulary is much bigger than the size of the atoms vocabulary in the MAT pretraining approach. The second task is a graph-level prediction proposed by Fabian et al. [15] in which the goal is to predict a set of real-valued descriptors of physicochemical properties. We present more detailed information about the pretraining procedure and ablations in Additional file 1.

**Other details.** Similarly to Maziarka et al. [12], we add an artificial dummy node to the input molecule. The distance of the dummy node to any other atom in the molecule is set to the maximal cutoff distance, and the edge connecting the dummy node with any other atom has its unique index. Moreover, the dummy node has its own index in the input atom embedding. We calculate distance information in a similar manner as Maziarka et al. [12]. The 3D molecular conformations that are used to obtain distance matrices are calculated using UFFOptimizeMolecule function from the RDKit package [47] with the default parameters. Finally, we consider a variant of the model extended with 200 RDKit features as in Rong et al. [16]. The features are concatenated to the final embedding ${\textbf{g}}$ and processed using a prediction MLP.

## Results and discussion

### Small hyperparameter budget

The drug discovery pipelines focus on fast iterations of compound screenings and adjusting the models to new data incoming from the laboratory. In particular, some approaches focus on the fast adaptation to the dataset by employing automated ML and reducing hands-on time [48]. We start by comparing in this setting R-MAT to DMPNN [17], MAT [12] and GROVER [16], representative state-of-the-art models on popular molecular property prediction tasks. We followed the evaluation in Maziarka et al. [12], where the only changeable hyperparameter is the learning rate, which was checked with 7 different values.

The BBBP and Estrogen-$\beta$ datasets use scaffold splits, while all the other datasets use random splits. Splits were proposed by Maziarka et al. [12]. For every dataset we calculate scores based on 6 different splits, we report the mean test score based on the hyperparameters that obtained the best validation score, in parentheses we include the standard deviation. In this and the next experiments, we denote models extended with additional RDKit features (see Section *Relative Molecule Self-Attention Transformer*) as $\hbox {GROVER}_{\text {rdkit}}$ and $\hbox {R-MAT}_{\text {rdkit}}$. More information about the models and datasets used in this benchmark is given in Additional file 1.

Table 3 shows that R-MAT outperforms other methods in 3 out of 6 tasks. For comparison, we also cite representative results of other methods from Maziarka et al. [12]. Satisfyingly, we observe a marked improvement on the solubility prediction tasks (ESOL and FreeSolv). Understanding solubility depends to a large degree on a detailed understanding of spatial relationships between atoms. This suggests that the improvement in performance might be related to better utilization of the distance or graph information.

**Table 3:** Results on molecule property prediction benchmark from Maziarka et al. [12]

	ESOL $\downarrow$	FreeSolv $\downarrow$	BBBP $\uparrow$	Estrogen-$\beta$ $\uparrow$	MetStab$_{\textrm{low}}$ $\uparrow$	MetStab$_{\textrm{high}}$ $\uparrow$
Pretrained models						
MAT	$0.278_{(0.020)}$	$0.265_{(0.042)}$	$0.737_{(0.009})$	$0.773_{(0.012)}$	$0.862_{( 0.025)}$	$0.884_{( 0.030)}$
GROVER	$0.303_{( 00.048)}$	$0.270_{( 0.033)}$	$0.726_{( 0.007)}$	$0.758_{( 0.006)}$	$0.892_{( 0.031)}$	$0.887_{( 0.019)}$
$\hbox {GROVER}_{\text {rdkit}}$	$0.288_{(0.021)}$	$0.308_{(0.058)}$	$0.726_{(0.003)}$	$0.788_{(0.009)}$	$0.873_{(0.033)}$	$0.881_{(0.039)}$
R-MAT	$0.252_{(0.030)}$	$\mathbf {0.232_{(0.071)}}$	$0.745_{(0.010)}$	$0.788_{(0.007)}$	$0.887_{(0.028)}$	$0.880_{(0.027)}$
$\hbox {R-MAT}_{\text {rdkit}}$	$\mathbf {0.246_{(0.024)}}$	$0.239_{(0.066)}$	$\mathbf {0.746_{(0.007)}}$	$0.791_{(0.010)}$	$0.884_{(0.032)}$	$0.886_{(0.031)}$
Non-pretrained models						
SVM	$0.479_{( 0.055)}$	$0.461_{( 0.077)}$	$0.723_{( 0.000)}$	$0.772_{( 0.000)}$	$0.893_{(0.030)}$	$\mathbf {0.890_{(0.029)}}$
$\hbox {SVM}_{\text {rdkit}}$	$0.279_{(0.024)}$	$0.285_{(0.049)}$	$0.741_{(0.001)}$	$0.781_{(0.001)}$	$0.895_{(0.029)}$	$0.884_{(0.031)}$
RF	$0.534_{( 0.073)}$	$0.524_{( 0.098)}$	$0.721_{( 0.003)}$	$0.791_{(0.012)}$	$0.892_{( 0.026)}$	$0.888_{( 0.030)}$
$\hbox {RF}_{\text {rdkit}}$	$0.289_{(0.035)}$	$0.337_{(0.026)}$	$0.743_{(0.002)}$	$\mathbf {0.807_{(0.003)}}$	$\mathbf {0.903_{(0.025)}}$	$0.886_{(0.028)}$
GCN	$0.369_{( 0.032)}$	$0.299_{( 0.068)}$	$0.695_{( 0.013)}$	$0.730_{( 0.006)}$	$0.884_{( 0.033)}$	$0.875_{( 0.036)}$
DMPNN	$0.297_{( 0.046)}$	$0.252_{( 0.044)}$	$0.709_{( 0.001)}$	$0.776_{( 0.006)}$	$0.885_{( 0.026)}$	$0.889_{( 0.018)}$

We only tune the learning rate for models in the first group. First two datasets are regression tasks (RMSE), other datasets are classification tasks (ROC AUC). For reference, we include results for non-pretrained baselines (SVM, RF, GCN [49], and DMPNN [17]) from [12]. We also include $\hbox {SVM}_{\text {rdkit}}$ and $\hbox {RF}_{\text {rdkit}}$ as two baseline methods with added RDKit features. The best results for each task are shown in bold. A rank plot for these experiments is included in Additional file 1

### Large hyperparameter budget

In contrast to the previous setting, we test R-MAT against a similar set of models but using a large-scale hyperparameter search (300 different hyperparameter combinations). This setting has been proposed in Rong et al. [16]. For comparison, we include results under small (7 different learning rates) hyperparameter budget. All datasets use a scaffold split. Scores are calculated based on 3 different data splits. While the ESOL and FreeSolv datasets are the same as in the previous paragraph, here they use a scaffold split, and the labels are not normalized (unlike in the previous paragraph). Additional information about the models and datasets used in this benchmark are given in Additional file 1.

Table 4 summarizes the experiment. The results show that for the large hyperparameter budget R-MAT outperforms other methods in 2 tasks and along with GROVER are the best in one more task. Overall in this setting our method achieves comparable results to GROVER, having the same median rank and being slightly worse in terms of mean rank. On the other hand, for small hyperparameters budget R-MAT achieves the best results, both in terms of the mean and the median ranks (see the details in Additional file 1).

**Table 4:** Results on the benchmark from Rong et al. [16]

	ESOL $\downarrow$	FreeSolv $\downarrow$	Lipo $\downarrow$	QM7 $\downarrow$	BACE $\uparrow$	BBBP $\uparrow$
Full hyperparameter tuning						
$\hbox {RF}_{\text {rdkit}}$	$0.942_{(0.196)}$	$2.625_{(0.509)}$	$0.739_{(0.038)}$	$124.3_{(3.5)}$	$\mathbf {0.884_{(0.030)}}$	$0.928_{(0.025)}$
GraphConv	$1.068_{(0.050)}$	$2.900_{(0.135)}$	$0.712_{(0.049)}$	$118.9_{(20.2)}$	$0.854_{(0.011)}$	$0.877_{(0.036)}$
Weave	$1.158_{(0.055)}$	$2.398_{(0.250)}$	$0.813_{(0.042)}$	$94.7_{(2.7)}$	$0.791_{(0.008)}$	$0.837_{(0.065)}$
DMPNN	$0.980_{(0.258)}$	$2.177_{(.914)}$	$0.653_{(0.046)}$	$105.8_{(13.2)}$	$0.852_{(0.053)}$	$0.919_{(0.030)}$
$\hbox {GROVER}_{\text {rdkit}}$	$0.888_{(0.116)}$	$\mathbf {1.592_{(0.072)}}$	$\mathbf {0.563_{(0.030)}}$	$72.5_{(5.9)}$	$0.878_{(0.016)}$	$\mathbf {0.936_{(0.008)}}$
$\hbox {R-MAT}_{\text {rdkit}}$	$\mathbf {0.786_{(0.133)}}$	$2.044_{(0.662)}$	$0.574_{(0.028)}$	$\mathbf {68.692_{(1.123)}}$	$.871_{(0.028)}$	$\mathbf {0.936_{(0.020)}}$
Learning rate only tuning						
MAT	$0.853_{(0.159)}$	$\underline{1.744_{(0.425)}}$	$0.608_{(0.017)}$	$102.8_{(2.94)}$	$0.846_{(0.025)}$	$0.920_{(0.039)}$
GROVER	$0.927_{(0.110)}$	$2.262_{(0.407)}$	$0.604_{(0.015)}$	$82.623_{(3.833)}$	$0.867_{(0.022)}$	$0.908_{(0.053)}$
$\hbox {GROVER}_{\text {rdkit}}$	$0.924_{(0.129)}$	$20.096_{(0.496)}$	$0.593_{(0.029)}$	$84.625_{(4.174)}$	$\underline{0.873_{(0.031)}}$	$\underline{0.931_{(0.021)}}$
R-MAT	$\underline{0.801_{(0.132)}}$	$1.912_{(0.364)}$	$0.585_{(0.029)}$	$77.248_{(2.819)}$	$0.858_{(0.041)}$	$\underline{0.931_{(0.016)}}$
$\hbox {R-MAT}_{\text {rdkit}}$	$0.819_{(0.145)}$	$2.057_{(0.434)}$	$\underline{0.580_{(0.019)}}$	$\underline{70.929_{(3.568)}}$	$0.858_{(0.021)}$	$0.920_{(0.021)}$

Models are fine-tuned under a large hyperparameters budget. Additionally, models fine-tuned with only tuning the learning rate are presented in the last group. The last two datasets are classification tasks (ROC AUC), the remaining datasets are regression tasks (MAE for QM7 and RMSE for the other datasets). For reference, we include results for non-pretrained baselines (GraphConv [50], Weave [51] and DMPNN [17]) from Rong et al. [16]. We also include $\hbox {RF}_{\text {rdkit}}$ as a baseline method with added RDKit features. A rank plot for these experiments is included in Additional file 1. The best scores for each task over all models are shown in bold, and the best scores for the models for which only the learning rate was tuned are underlined

### Large-scale experiments

Finally, to better understand how R-MAT performs in a setting where pretraining is likely to less influence results, we include results on the QM9 dataset [52]. QM9 is a quantum mechanics benchmark that encompasses the prediction of 12 simulated properties across around 130 k small molecules with at most 9 heavy (non-hydrogen) atoms. The molecules are provided with their atomic 3D positions for which the quantum properties were initially calculated. For these experiments, we used a learning rate equal to 0.015 (we selected this learning rate value as it returned the best results for $\alpha$ dataset among 4 different learning rates that we tested: {0.005,0.01,0.015,0.02}). We present additional information about the dataset and models used in this benchmark in Additional file 1.

**Fig. 4:**
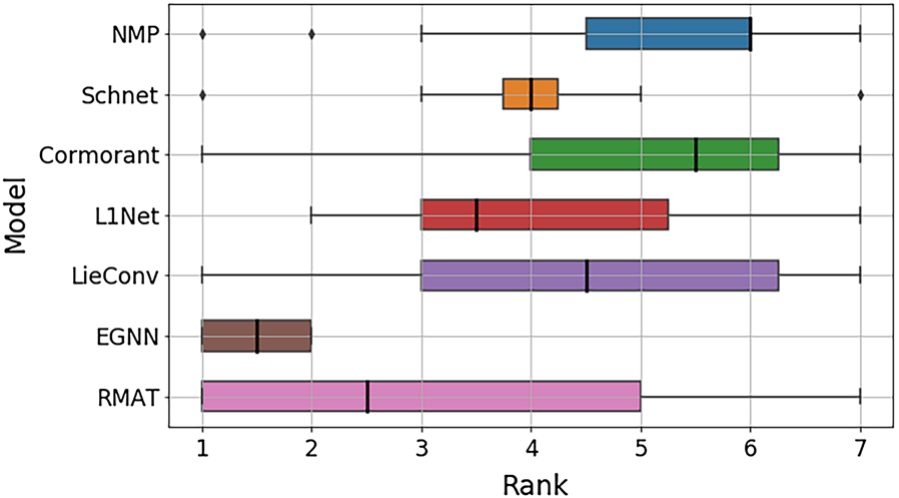
Rank plot of scores obtained on the QM9 benchmark, which consists of 12 different quantum property prediction tasks

Figure 4 compares R-MAT performance with various models. More detailed results could be found in Additional file 1. R-MAT achieves highly competitive results, with state-of-the-art performance on 4 out of the 12 tasks. We attribute higher variability of performance to the limited small hyperparameter search we performed.

### Exploring the design space of self-attention layer

Achieving strong empirical results hinged on a methodological exploration of the design space of different variants of the self-attention layer. We document here this exploration and relevant ablations. We present here the experiments for different relative attention features and different choices of maximum neighborhood order. We also defer most results to the Additional file 1, where we present experiments for different self-attention variants, distance encoding and bond features. We perform all experiments on the ESOL, FreeSolv, and BBBP datasets with 3 different scaffold splits. We did not use any pretraining for these experiments. We follow the same fine-tuning methodology as in Section *Small hyperparameter budget*.

**Table 5:** Ablations of relative molecule self-attention; other ablations are included in the Additional file 1

	BBBP $\uparrow$	ESOL $\downarrow$	FreeSolv $\downarrow$
(a) Test set performances of R-MAT for different relative attention features.			
R-MAT	$0.872_{(0.042)}$	$0.400_{(0.044)}$	$0.430_{(0.056)}$
Distance	$0.877_{(0.062)}$	$0.407_{(0.037)}$	$0.484_{(0.037)}$
Neighborhood	$0.872_{(0.055)}$	$0.402_{(0.027)}$	$0.493_{(0.046)}$
Bond features	$0.871_{(0.057)}$	$0.403_{(0.026)}$	$0.460_{(0.025)}$
Only distance	$0.870_{(0.038)}$	$0.418_{(0.036)}$	$0.504_{(0.072)}$
Only neighborhood	$0.886_{(0.038)}$	$0.406_{(0.032)}$	$0.483_{(0.042)}$
Only bond features	$0.894_{(0.049)}$	$0.407_{(0.034)}$	$0.494_{(0.018)}$

	BBBP $\uparrow$	ESOL $\downarrow$	FreeSolv $\downarrow$
(b) Test set performances of R-MAT for different choices of maximum neighborhood order.			
R-MAT	$0.908_{(0.039)}$	$0.378_{(0.027)}$	$0.438_{(0.036)}$
Max order = 1	$0.847_{(0.081)}$	$0.372_{(0.018)}$	$0.461_{(0.049)}$
Max order = 2	$0.890_{(0.068)}$	$0.382_{(0.040)}$	$0.519_{(0.036)}$
Max order = 3	$0.873_{(0.053)}$	$0.455_{(0.005)}$	$0.492_{(0.055)}$

**Importance of different sources of information in self-attention.** The self-attention module in R-MAT incorporates three auxiliary sources of information: (1) distance information, (2) graph information (encoded using neighborhood order), and (3) bond features. In Table 5(a), we show the effect on the performance of ablating each of these elements. In this experiment, we repeat the calculations for three different data splits and five different random seeds to make the results less prone to random noise, e.g. due to the random weight initialization. We find that all components, including the distance matrix, are crucial for achieving optimal performance of R-MAT. The use of all information sources results in the best performance across all tested datasets. The performance for the smallest FreeSolv dataset is considerably better when more information sources are included. The same trend is observed in the larger ESOL regression task, albeit with less noticeable differences. For the BBBP binary classification task, all results seem comparable, but interestingly, all variants without inter-atomic distances achieve better results.

**Maximum neighborhood order.** We take a closer look at how we encode the molecular graph. Maziarka et al. [12] used a simple binary adjacency matrix to encode the edges. We enriched this representation by adding one-hot encoding of the neighborhood order. For example, the order of 3 for a pair of atoms means that there are two other vertices on the shortest path between this pair of atoms. In R-MAT we used 4 as the maximum order of neighborhood distance. That is, we encoded as separate features if two atoms are 1, 2, 3 or 4 *hops* away in the molecular graph. In Table 5 (b) we ablate this choice. The result suggests that R-MAT performance benefits from including separate features for all the considered orders.

### Closer comparison to molecule attention transformer

Our main motivation for improving self-attention in MAT was to make it easier to represent attention patterns that depend in a more complex way on the distance and graph information. We qualitatively explore here whether R-MAT achieves this goal, comparing its attention patterns to that of MAT.

**Fig. 5:**
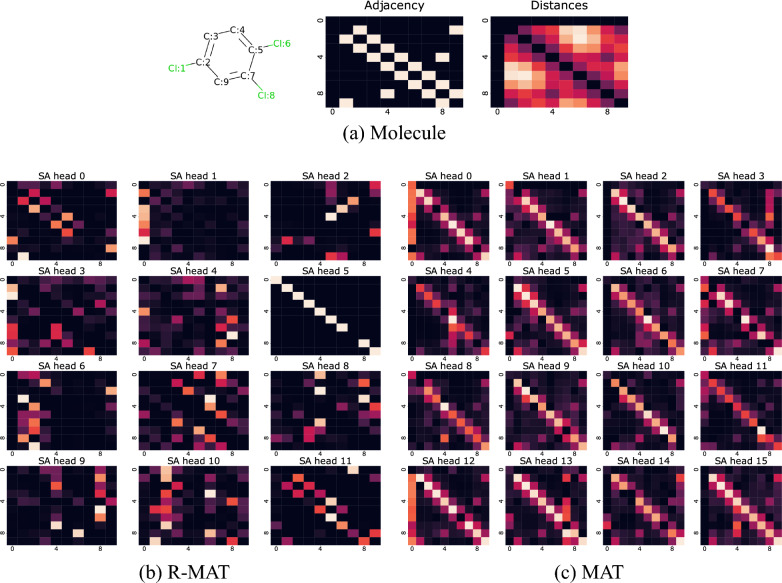
Visualization of the learned self-attention for each of all attention heads in the second layer of pretrained R-MAT (left) and all attention heads in pretrained MAT (right), for a molecule from the ESOL dataset. The top Figure visualizes the molecule and its adjacency and distance matrices. The self-attention pattern in MAT is dominated by the adjacency and distance matrix, while R-MAT seems capable of learning more complex attention patterns

We compared attention patterns learned by the pretrained MAT (weights obtained from Maziarka et al. [12]) and R-MAT. We observed that long-range atom relations are better captured by our model. We demonstrate this finding for a selected molecule from the ESOL dataset. Figure 5 shows that different heads of Relative Molecule Self-Attention are focusing on different atoms in the input molecule. We can see that self-attention strength is concentrated on the input atom (head 5), on the closest neighbors (heads 0 and 11), on the second-order neighbors (head 7), on the dummy node (head 1) or on some substructure that occurs in the molecule (heads 6 and 10 are concentrated on atoms 1 and 2). In contrast, self-attention in MAT focuses mainly on the input atoms and their closest neighbors, the information from other regions of the molecule is not strongly propagated. This likely happens due to the construction of the Molecule Self-Attention in MAT (c.f. Eq. (2)), where the output atom representation is calculated from equally weighted messages based on the adjacency matrix, distance matrix, and self-attention. Due to its construction, it is more challenging for MAT than for R-MAT to learn to attend to a distant neighbor.

As Relative Molecule Self-Attention Transformer is an extension of Molecule Attention Transformer [12], we perform a more strict comparison of these models. To this end, we compare MAT with R-MAT using three different pretraining strategies: no pretraining, masking pretraining (following the original MAT model), and contextual + graph level pretraining (presented in this paper). For this comparison, we use the small hyperparameter budget benchmarks used in the MAT paper (and in this paper, in Section *Small hyperparameter budget*).

The results of the comparison between MAT and R-MAT are presented in Table 6. R-MAT, on average, obtains better results than the standard MAT. Moreover, the more complicated the pretraining is, the better R-MAT is compared to MAT. In the case of no pretraining, R-MAT outperforms MAT on 3 out of 6 tasks, the scores for one task are equal, and R-MAT is outperformed by MAT on 2 out of 6 tasks. In the case of the masked pretraining, R-MAT achieves better scores, outperforming MAT on 4 out of 6 tasks. Finally, in the contextual + graph level pretraining setting, R-MAT outperforms MAT on 5 out of 6 tasks.

**Table 6:** Results of the direct comparison between R-MAT and MAT, for different pre-training settings

		ESOL $\downarrow$	FreeSolv $\downarrow$	BBBP $\uparrow$	Estrogen-$\beta$ $\uparrow$	MetStab$_{\textrm{low}}$ $\uparrow$	MetStab$_{\textrm{high}}$ $\uparrow$
No pretraining	MAT	$0.278_{( 0.019)}$	$0.283_{(0.043)}$	$\underline{0.727_{(0.008)}}$	$0.751_{(0.005)}$	$\underline{0.857_{(0.025)}}$	$\underline{0.872_{( 0.051)}}$
	R-MAT	$\underline{0.273_{(0.046)}}$	$\underline{0.272_{(0.015)}}$	$\underline{0.727_{(0.015)}}$	$\underline{0.786_{(0.014)}}$	$0.844_{(0.050)}$	$0.833_{( 0.042)}$
Masking pretraining	MAT	$0.278_{( 0.020)}$	$0.265_{( 0.042)}$	$\underline{0.737_{( 0.009)}}$	$0.773_{( 0.012)}$	$0.862_{( 0.025)}$	$\underline{0.884_{( 0.030)}}$
	R-MAT	$\underline{0.253_{(0.085)}}$	$\underline{0.264_{(0.028)}}$	$0.714_{(0.090)}$	$\underline{0.789_{(0.015)}}$	$\underline{0.880_{(0.022)}}$	$0.870_{(0.042)}$
R-MAT pretraining	MAT	$0.298_{(0.024)}$	$0.246_{(0.042)}$	$0.729_{(0.006)}$	$0.782_{(0.021)}$	$0.879_{(0.024)}$	$\underline{0.882_{( 0.030)}}$
	R-MAT	$\underline{0.252_{(0.030)}}$	$\underline{0.232_{(0.071)}}$	$\underline{0.745_{(0.010)}}$	$\underline{0.788_{(0.007)}}$	$\underline{0.887_{(0.028)}}$	$0.880_{(0.027)}$

We underline the best scores for every pretraining setting

### Limitations

Although R-MAT has shown promising results, there are a few limitations to our approach that should be considered. Firstly, our model is E(3)-invariant thanks to the use of inter-atomic distances, but it lacks the ability to recognize mirror images (enantiomers), which might be crucial for some tasks such as binding affinity prediction. Secondly, our model uses only one sampled molecular conformation for the prediction, thereby missing out on the entire range of other possible conformations for molecules that are highly flexible. Finally, our model is currently limited to predicting properties for small to medium-sized molecules and may not be suitable for larger, more complex molecules. R-MAT, like many other transformers, is computationally intensive and requires memory quadratic with the input molecule size. These limitations provide opportunities for future research to address these challenges and improve upon our results.

## Conclusions

Transformer has been successfully adapted to various domains by incorporating into its architecture a minimal set of inductive biases. In a similar spirit, we methodologically explored the design space of the self-attention layer and identified a highly effective Relative Molecule Self-Attention.

Relative Molecule Self-Attention Transformer, a model based on Relative Molecule Self-Attention, achieves state-of-the-art or very competitive results across a wide range of molecular property prediction tasks. R-MAT is a highly versatile model, showing competitive results in both quantum property prediction tasks, as well as on biological datasets. We also show that R-MAT is easy to train and requires tuning only the learning rate to achieve competitive results, which together with open-sourced weights and code, makes our model highly accessible.

Relative Molecule Self-Attention encodes an inductive bias to consider relationships between atoms that are commonly relevant to a chemist, but on the other hand, leaves flexibility to unlearn them if needed. Relatedly, Vision Transformers learn global processing in early layers despite being equipped with a locality inductive bias [18]. Our empirical results show in a new context that picking the right set of inductive biases is key for self-supervised learning to work well. We also show that Relative Molecule Self-Attention will help improve other models for molecular property prediction.

Learning useful representations for molecular property prediction is far from being solved. Achieving state-of-the-art results, while less dependent on them, still relied on using certain large sets of handcrafted features both in fine-tuning and pretraining. At the same time, these features are beyond doubt learnable from data. Developing methods that will push representation learning towards discovering these and better features automatically from data is an exciting challenge for the future.

### Supplementary Information

**Additional file 1.** Additional experiments, supplementary tables and figures.

## Data Availability

We open-source R-MAT weights and code as part of the HuggingMolecules package [53] at: https://github.com/gmum/huggingmolecules. We also share all datasets and data splits that we used in our experiments at: https://osf.io/rgva4/.

## Footnotes

1 This arises for example when input is an arbitrary chunk of the text [22] (e.g. in the next sentence prediction task used in BERT pretraining).
