# Supplementary material for: Relative molecule self-attention transformer
Source: J Cheminform. 2024 Jan 3;16:3. doi: 10.1186/s13321-023-00789-7 (PMC10765783; doi:10.1186/s13321-023-00789-7)
Supplement: Supplementary file 1 — Additional file 1. Additional experiments, supplementary tables and figures. [file 13321_2023_789_MOESM1_ESM.pdf]

Supporting Information for
Relative Molecule Self-Attention Transformer

Appendix A: R-MAT node features

The input molecule is embedded as a matrix of size $N_{\text{atom}} \times 36$ where each atom of the input is embedded following Coley et al. [1] and Pocha et al. [2]. All features are presented in Table A.1.

Table A.1: Featurization used to embed atoms in R-MAT.

Indices	Description
$0-11$	Atomic identity as a one-hot vector of B, N, C, O, F, P, S, Cl, Br, I, Dummy, other
$12-17$	Number of heavy neighbors as one-hot vector of 0, 1, 2, 3, 4, 5
$18-22$	Number of hydrogen atoms as one-hot vector of 0, 1, 2, 3, 4
$23-33$	Formal charge as one-hot vector of -5, -4, ..., 4, 5
34	Is in a ring
35	Is aromatic

Appendix B: Pretraining

We extend the pretraining procedure proposed by Maziarka et al. [3], who used a masking task based on [4, 5]; they masked types of some of the graph atoms and treat them as the label, that should be predicted by the neural network. Such an approach works well in NLP where models pretrained with the masking task create the state-of-the-art representation [4, 6, 7]. However in chemistry, otherwise than in NLP, the size of atoms vocabulary is much smaller. Moreover, usually only one type of atom fits a given place and thus the representation trained with the masking task has problems with encoding meaningful information in chemistry.

B.1 Contextual pretraining

Instead of atom masking, we used a two-step pretraining that combines the procedures proposed by Rong et al. [8] and Fabian et al. [9]. In the first step, the network is trained with the contextual property prediction task [8], where we mask not only the selected atoms but also their neighbors. The task is then to predict the whole atom context, e.g. if the selected atom's type is carbon connected with a nitrogen with a double bond and with an oxygen with a single bond, we encode the atom neighborhood as C_N-DOUBLE1_O-SINGLE1 (we list all the node-edge counts terms in the alphabetical order), then the network has to predict the specific type of the masked neighborhood for every masked atom. This task is much more demanding

for the network than the classical masking approach presented by Maziarka et al. [3] as the network has to encode more specific information about the masked atom's neighborhood. Furthermore, the size of context vocabulary is much bigger than the size of atoms vocabulary in the MAT pretraining approach (2925 for R-MAT vs 35 for MAT).

B.2 Graph-level pretraining

The second task is the graph-level property prediction proposed by Fabian et al. [9]. In this pretraining procedure, the task is to predict 200 real-valued descriptors of physicochemical characteristics of every given molecule.

The list of all 200 descriptors from RDKit is as follows:
`BalabanJ, BertzCT, Chi0, Chi0n, Chi0v, Chi1, Chi1n, Chi1v, Chi2n, Chi2v, Chi3n, Chi3v, Chi4n, Chi4v, EState_VSA1, EState_VSA10, EState_VSA11, EState_VSA2, EState_VSA3, EState_VSA4, EState_VSA5, EState_VSA6, EState_VSA7, EState_VSA8, EState_VSA9, ExactMolWt, FpDensityMorgan1, FpDensityMorgan2, FpDensityMorgan3, FractionCSP3, HallKierAlpha, HeavyAtomCount, HeavyAtomMolWt, Ipc, Kappa1, Kappa2, Kappa3, LabuteASA, MaxAbsEStateIndex, MaxAbsPartialCharge, MaxEStateIndex, MaxPartialCharge, MinAbsEStateIndex, MinAbsPartialCharge, MinEStateIndex, MinPartialCharge, MolLogP, MolMR, MolWt, NHOHCount, NOCount, NumAliphaticCarbocycles, NumAliphaticHeterocycles, NumAliphaticRings, NumAromaticCarbocycles, NumAromaticHeterocycles, NumAromaticRings, NumHAcceptors, NumHDonors, NumHeteroatoms, NumRadicalElectrons, NumRotatableBonds, NumSaturatedCarbocycles, NumSaturatedHeterocycles, NumSaturatedRings, NumValenceElectrons, PEOE_VSA1, PEOE_VSA10, PEOE_VSA11, PEOE_VSA12, PEOE_VSA13, PEOE_VSA14, PEOE_VSA2, PEOE_VSA3, PEOE_VSA4, PEOE_VSA5, PEOE_VSA6, PEOE_VSA7, PEOE_VSA8, PEOE_VSA9, RingCount, SMR_VSA1, SMR_VSA10, SMR_VSA2, SMR_VSA3, SMR_VSA4, SMR_VSA5, SMR_VSA6, SMR_VSA7, SMR_VSA8, SMR_VSA9, SlogP_VSA1, SlogP_VSA10, SlogP_VSA11, SlogP_VSA12, SlogP_VSA2, SlogP_VSA3, SlogP_VSA4, SlogP_VSA5, SlogP_VSA6, SlogP_VSA7, SlogP_VSA8, SlogP_VSA9, TPSA, VSA_EState1, VSA_EState10, VSA_EState2, VSA_EState3, VSA_EState4, VSA_EState5, VSA_EState6, VSA_EState7, VSA_EState8, VSA_EState9, fr_Al_COO, fr_Al_OH, fr_Al_OH_noTert, fr_ArN, fr_Ar_COO, fr_Ar_N, fr_Ar_NH, fr_Ar_OH, fr_COO, fr_COO2, fr_C_O, fr_C_O_noCOO, fr_C_S, fr_HOCCN, fr_Imine, fr_NH0, fr_NH1, fr_NH2, fr_N_O, fr_Ndealkylation1, fr_Ndealkylation2, fr_Nhpyrrole, fr_SH, fr_aldehyde, fr_alkyl_carbamate, fr_alkyl_halide, fr_allylic_oxid, fr_amide, fr_amidine, fr_aniline, fr_aryl_methyl, fr_azide, fr_azo, fr_barbitur, fr_benzene, fr_benzodiazepine, fr_bicyclic, fr_diazo, fr_dihydropyridine, fr_epoxide, fr_ester, fr_ether, fr_furan, fr_guanido, fr_halogen, fr_hdrzine, fr_hdrzone, fr_imidazole, fr_imide, fr_isocyan, fr_isothiocyan, fr_ketone, fr_ketone_Topliss, fr_lactam, fr_lactone, fr_methoxy, fr_morpholine, fr_nitrile, fr_nitro, fr_nitro_arom, fr_nitro_arom_nonortho, fr_nitroso, fr_oxazole, fr_oxime, fr_para_hydroxylation, fr_phenol, fr_phenol_noOrthoHbond, fr_phos_acid, fr_phos_ester, fr_piperdine, fr_piperzine, fr_priamide, fr_prisulfonamd, fr_pyridine, fr_quatN, fr_sulfide, fr_sulfonamd, fr_sulfone, fr_term_acetylene, fr_tetrazole, fr_thiazole, fr_thiocyan, fr_thiophene, fr_unbrch_alkane, fr_urea, qed`

Appendix C: Experimental setting

C.1 Model hyperparameters

R-MAT model consists of 10 layers with 12 attention heads in each, $d_{model} = 768$. The distance layer consists of 32 radial functions ($N_{emb} = 32$) and cutoff distance c is set to 20 Å. Attention pooling consists of 4 pooling heads and pooling hidden dimension is set to 128. Prediction MLP consists of one hidden layer with dimension set to 1024 and dropout 0.1. R-MAT uses leaky-ReLU with slope 0.1 as a non-linearity in all experiments. This set of hyperparameters defines a model with 48 million of parameters, which is equal to the number of parameters of $\text{GROVER}_{\text{base}}$ and is slightly higher than the number of parameters of MAT (42M).

C.2 3D conformations

The 3D molecular conformations that are used to obtain distance matrices were calculated using UFFOptimizeMolecule function from the RDKit package [10] with the default parameters (maxIters=200, vdwThresh=10.0, confId=−1, ignoreInterfragInteractions=True).

One disadvantage of this approach is the costly calculation of the distance matrix. This is not burdensome for small datasets with small molecules (e.g FreeSolv), however, they can be a problem with larger ones (e.g. dataset for pretraining). In Table C.2 we present dataset sizes and molecular statistics for three different datasets (FreeSolv, ESOL, BBBP), as well as the time needed for molecular conformations calculation. In Figure C.1 we present these times for every single molecule from these datasets. Based on these results one can see, that the larger the molecule, the longer the conformation calculation time. Moreover, even for BBBP, which is not rather a large dataset, calculating the conformations could take almost 5 minutes. We leave exploring other conformation methods (more accurate or faster) as an interesting topic for the future.

Table C.2: Time needed for molecular conformations calculation for different datasets.

	Dataset size	Average number of atoms	Calculation time (s)	Average calculation time (s)
FreeSolv	622	8.72	5.225	0.008
ESOL	1128	13.28	27.865	0.024
BBBP	2037	24.02	267.084	0.131

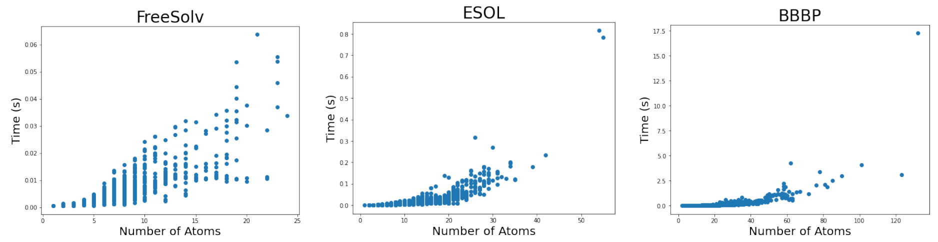

Figure C.1: Time needed for conformations calculation for every single molecule for different datasets.

C.3 Pretraining

We pretrained R-MAT on 4 million of unlabelled molecules. Molecules were obtained from the ZINC15 [11] and ChEMBL [12] datasets, by first taking a sample of 10M molecules and then filtering them using the Lipinski's rule of five [13]. We have split the data into training and validation datasets, where the validation dataset consists of 5% of the data. We pretrained R-MAT for 150 epochs. We used dropout equal to 0.1, learning rate 0.001, the Noam optimizer [14] trained with 20000 warm-up steps and batch size 256. We pretrained R-MAT with 8 Nvidia A100 GPUs, using the Horovod package [15].

Overlap between pre-trained and task datasets In Table C.3 we provide information about the number of atoms that overlaps between pre-trained dataset and all tasks datasets used in this paper. One can see that usually the overlap is at most a few percent of all dataset molecules. This overlap does not affect the test performance, as pre-training consists of a semi-supervised task of context prediction and another task, associated with the prediction of simple physico-chemical characteristics, loosely related to the fine-tuning tasks, which are additionally added to the final graph embedding during the prediction.

Table C.3: Overlap between pre-trained dataset and different tasks datasets.

dataset	overlap molecules	overlap percentage
bbbp	34	1.6%
bace	0	0.0%
esol	83	7.3%
estrogen-beta	45	2.2%
freesolv	2	0.3%
lipo	128	3.0%
mesta-high	608	28.5%
mesta-low	608	28.5%
qm7	0	0.0%
qm9	0	0.0%

C.4 Small hyperparameter budget

Models We compared R-MAT with four models trained from scratch: Support Vector Machine with RBF kernel (SVM) and Random Forest (RF) that both works on ECFP fingerprints [16], Graph Convolutional Network [17] (GCN) and Directed Message Passing Neural Network [18] (DMPNN).

The comparison also includes two different pretrained models: MAT [3] and GROVER [8].

Datasets The benchmark is based on important molecule property tasks in the drug discovery domain. The first two datasets are ESOL and FreeSolv, in which the task is to predict the solubility of a molecule in water – a key property of any drug – and the error is measured using RMSE. The goal in BBBP and Estrogen$-\beta$ is to classify correctly whether a given molecule is active against a biological target. For details on other tasks please see Maziarka et al. [3]. BBBP and Estrogen$-\beta$ used scaffold split, rest datasets used the random split method. For every dataset 6 different splits were created. Labels of regression datasets (ESOL and FreeSolv)

were normalized before training. We did not include the Estrogen$-\alpha$ dataset that was also presented by Maziarka et al. [3], due to the GPU memory limitations (as the biggest molecule from this dataset consists of over 500 atoms).

Training hyperparameters We fine-tune R-MAT on the target tasks for 100 epochs, with batch size equal to 32 and Noam optimizer with warm-up equal to 30% of all steps. The only hyperparameter that we tune is the learning rate, which is selected from the set of 7 possible options: $\{1e{-}3, 5e{-}4, 1e{-}4, 5e{-}5, 1e{-}5, 5e{-}6, 1e{-}6\}$. This small budget for hyperparameter selection reflects the long-term goal of this paper of developing easy-to-use models for molecule property prediction. The fine-tuning was conducted using Nvidia V100 GPU.

C.5 Large hyperparameter budget

Models For large hyperparameters budget we compared R-MAT with three models trained from scratch: GraphConv [19], Weave [20] and DMPNN [18] and two pretrained models: MAT [3] and GROVER [8]. For small hyperparameters budget, we compared R-MAT to MAT and GROVER. We note that R-MAT, MAT and GROVER use different pretraining methods. MAT was pretrained with 2M molecules from the ZINC database and GROVER was pretrained with 10M molecules from the ZINC and ChEMBL databases.

Datasets All datasets were split using a scaffold split. The resulting splits are different than in the MAT benchmark. For every dataset, 3 different splits were created. In our comparison, we included only the subset of single-task datasets from the original GROVER work [8]. This is the reason why we use a smaller number of datasets. The obtained regression scores for ESOL differ significantly from the small hyperparameters budget benchmark because this time labels are not normalized.

Training hyperparameters For learning rate tuning we used the same hyperparameters settings as in the MAT benchmark (see the *Small hyperparameter budget* section above).

For large hyperparameters budget, we run a random search with the hyperparameters listed in Table C.4.

Table C.4: Relative Molecule Self-Attention Transformer large grid hyperparameters ranges

	parameters
warmup	0.05, 0.1, 0.2, 0.3
learning rate	0.005, 0.001, 0.0005, 0.0001, 0.00005, 0.00001, 0.000005, 0.000001
epochs	100
pooling hidden dimension	64, 128, 256, 512, 1024
pooling attention heads	2, 4, 8
prediction MLP layers	1, 2, 3
prediction MLP dim	256, 512, 1024, 2048
prediction MLP dropout	0.0, 0.1, 0.2

C.6 Large-scale experiments

Models We compared our R-MAT with 8 different models: NMP [21], Schnet [22], Cormorant [23], L1Net [24], LieConv [25], TFN [26], SE(3)-Tr. [27], EGNN [28].

Datasets The QM9 dataset [29] is a dataset that consists of molecules, with up to 9 heavy atoms (H, C, N, O, F) and up to 29 atoms overall per molecule. Each atom from this dataset is additionally associated with 3D position. The dataset consists of 12 different regression tasks named: α, $\Delta\varepsilon$, $\varepsilon_{\mathrm{HOMO}}$, $\varepsilon_{\mathrm{LUMO}}$, μ, C_ν, G, H, R^2, U, U_0, ZPVE, for which the mean absolute error is a standard metric. The dataset has over 130k molecules. We use data splits proposed by Anderson et al. [23], which gives us 100k training molecules, 18k molecules for validation and 13k molecules for testing.

Training hyperparameters We trained R-MAT for 1000 epochs, with batch size equal to 256 and learning rate equal to 0.015. We report the test set MAE for the epoch with the lowest validation MAE. We selected this learning rate value as it returned the best results for α among 4 different learning rates that we tested: $\{0.005, 0.01, 0.015, 0.02\}$.

C.7 Ablations

Datasets For the ablations section, we used the BBBP, ESOL and FreeSolv datasets, split using a scaffold split, with 3 different splits. Labels of the regression datasets (ESOL and FreeSolv) were normalized before training. Scores obtained in this section differ significantly from the previous benchmarks due to the different data splits, different model hyperparameters and no pretraining used.

Training hyperparameters Similarly as for our main benchmarks, we tuned only the learning rate, which was selected from the set of 7 possible options: $\{1e{-}3, 5e{-}4, 1e{-}4, 5e{-}5, 1e{-}5, 5e{-}6, 1e{-}6\}$. We used batch size equal to 32 and Noam optimizer with warm-up equal to 20% of all steps. Moreover, we use a single-layer, instead of a two-layer, MLP as our classification part.

Appendix D: Additional experimental results

D.1 Small hyperparameter budget

In Figure D.2 one can find rank plots for the results obtained using a small hyperparameter budget. R-MAT and R-MAT $_{\mathrm{rdkit}}$ obtained the best median rank among all compared models. The performance gain of $\mathrm{RF}_{\mathrm{rdkit}}$ over RF and $\mathrm{SVM}_{\mathrm{rdkit}}$ over SVM is interesting. Overall these two baseline models are worse only than R-MAT.

D.2 Large hyperparameter budget

In Figure D.3 one can find rank plots for the results using a large hyperparameter budget. We present separate plots for models trained with the large grid search (Left) and for models with only learning rate tuning (Right).

D.3 Large-scale experiments

In Table D.5 one can find detailed results of comparison R-MAT performance with other various models. R-MAT achieves highly competitive results, with state-of-the-art performance on 4 out of the 12 tasks, which proves how universal this model is.

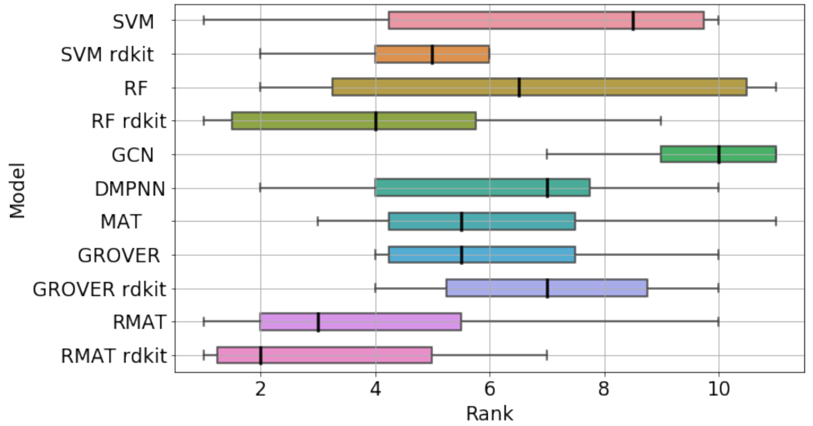

Figure D.2: Rank plot for small hyperparameter budget experiments.

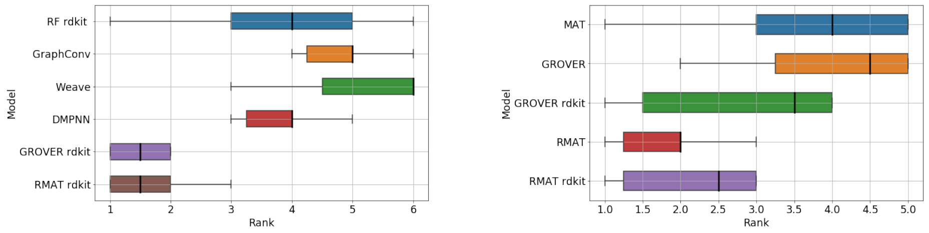

(a) Large grid-search

(b) Learning rate only grid-search

Figure D.3: Rank plot for large hyperparameter budget (Left) as well as for models trained with only the learning rate tunning (Right).

Appendix E: Exploring the design space of molecular self-attention

Identifying the Relative Molecule Self-Attention layer required a large-scale and methodological exploration of the self-attention design space. In this section, we present experimental data that informed our choices. We also hope it will inform future efforts in designing attention mechanism for molecular data. We follow here the same evaluation protocol as in the ablation study and show how different natural variants compare against R-MAT.

E.1 Self-attention variants

Relative Molecule Self-Attention is designed to better incorporate the relative spatial position of atoms in the molecule. The first step is embedding each pair of atoms. Then, the embedding is used to re-weight self-attention. To achieve this, Relative Molecule Self-Attention combines ideas from natural language processing [30, 31, 32]. These works focus on encoding better relative positions of tokens in the input.

We compare to three specific variants from these works that can be written using our previously introduced notation as:

Table D.5: Mean absolute error on QM9, a benchmark including various quantum prediction tasks. Results are cited from the literature.

Task<br>Units	α<br>bohr^3	$\Delta\varepsilon$<br>meV	$\varepsilon_{\text{HOMO}}$<br>meV	$\varepsilon_{\text{LUMO}}$<br>meV	μ<br>D	C_ν<br>cal/mol K	G<br>meV	H<br>meV	R^2<br>bohr^3	U<br>meV	U_0<br>meV	ZPVE<br>meV
NMP	.092	69	43	38	.030	.040	19	17	.180	20	20	1.50
Schnet	.235	63	41	34	.033	.033	14	14	.073	19	14	1.70
Cormorant	.085	61	34	38	.038	.026	20	21	.961	21	22	2.03
L1Net	.088	68	46	35	.043	.031	14	14	.354	14	13	1.56
LieConv	.084	49	30	25	.032	.038	22	24	.800	19	19	2.28
TFN	.223	58	40	38	.064	.101	-	-	-	-	-	-
SE(3)-Tr.	.142	53	35	33	.051	.054	-	-	-	-	-	-
EGNN	.071	48	29	25	.029	.031	12	12	.106	12	11	1.55
R-MAT $_{\text{rdkit}}$	.082	48	31	29	.110	.036	10	10	.676	10	12	2.23

1 Relative self attention [30]:

$$e_{ij} = (x_i W^Q)(x_j W^K)^T + (x_i W^Q)\boldsymbol{b}_{ij}^K.$$

2 Relative self attention with attentive bias [31]:

$$e_{ij} = (x_i W^Q)(x_j W^K)^T + (x_i W^Q)\boldsymbol{b}_{ij}^K + \boldsymbol{u}^T(x_j W^K) + \boldsymbol{v}^T\boldsymbol{b}_{ij}^K.$$

3 Improved relative self-attention [32]:

$$e_{ij} = (x_i W^Q)(x_j W^K)^T + (x_i W^Q)\boldsymbol{b}_{ij}^K + (x_j W^K)\boldsymbol{b}_{ij}^K.$$

Table E.6 shows that the attention operation used in R-MAT outperforms variants 2 and 3 across all three tasks and variant 1 on two tasks, being comparable on the third one. This might be expected given that Relative Molecule Self-Attention combines these ideas.

Table E.6: Test set performances of R-MAT for different choices of the relative self-attention.

	BBBP	ESOL	FreeSolv
R-MAT	$.908_{(.039)}$	$.378_{(.027)}$	$.438_{(.036)}$
Relative type = 1	$.859_{(.057)}$	$.371_{(.041)}$	$.509_{(.028)}$
Relative type = 2	$.856_{(.049)}$	$.424_{(.014)}$	$.472_{(.057)}$
Relative type = 3	$.882_{(.051)}$	$.389_{(.040)}$	$.441_{(.021)}$

E.2 Enriching bond features with atom features

In Relative Molecule Self-Attention, we use a small number of bond features to construct the atom pair embedding. We investigate here the effect of extending bond featurization.

Inspired by Shang et al. [33], we added information about the atoms that an edge connects. We tried three different variants. In the first one, we extend the bond representation with concatenated input features of atoms that the bond connects. In the second one, instead of raw atoms' features, we tried the one-hot-encoding of the type of the bond connection (i.e. when the bond connects atoms C and N,

Table E.7: Test set performances of R-MAT for different choices of bond featurization.

	BBBP	ESOL	FreeSolv
R-MAT	$.908_{(.039)}$	$.378_{(.027)}$	$.438_{(.036)}$
Connected atoms features	$.866_{(.073)}$	$.406_{(.048)}$	$.489_{(.046)}$
Connection type one-hot	$.863_{(.012)}$	$.411_{(.028)}$	$.510_{(.055)}$
Both	$.873_{(.034)}$	$.390_{(.020)}$	$.502_{(.044)}$

we encode it as a bond 'C_N' and take the one-hot-encoding of this information). Finally, we combined these two approaches together.

The results are shown in Table E.7. Surprisingly, we find that adding this type of information to the bond features negatively affects the performance of R-MAT. This suggests that R-MAT can already access these features efficiently from the input (which we featurize using the same set of features). This could also happen due to the fact that after a few layers, the attention is not calculated over the input atoms anymore. Instead, it works over hidden embeddings, which themselves can be mixed representations of multiple atom embeddings [34], where the proposed additional representation contains only information about the input features.

E.3 Distance encoding variants

R-MAT uses a specific radial base distance encoding proposed by Klicpera et al. [35], followed by the envelope function, with $N_{emb} = 32$. We compare here to several other natural choices.

We tested the following distance encoding variants : (1) removal of the envelope function, (2) increasing the number of distance radial functions to 128, (3) using distance embedding from the popular MAT model [3], (4) using distance embedding from the popular SchNet model [22]. The distance in MAT is encoded as $e(d) = \exp(-d)$. The distance in SchNet is encoded as $e_n(d) = \exp(-\gamma \|d - \mu_n\|^2)$, for $\gamma = 10\text{Å}$ and $0\text{Å} \leq \mu_n \leq 30\text{Å}$ divided into $\mathrm{N_{emb}}$ equal sections, with $\mathrm{N_{emb}}$ set to 32 or 128.

The results are shown in Table E.8. These results corroborate that a proper representation of distance information is a key in adapting self-attention to molecular data. We observe that all variants underperform compared to the radial base encoding used in Relative Molecule Self-Attention. Noteworthy is the fact that encoding distances in MAT way caused a lot of instability during the training of R-MAT.

Table E.8: Test set performances of R-MAT for different choices of distance modelling.

	BBBP	ESOL	FreeSolv
R-MAT	$.908_{(.039)}$	$.378_{(.027)}$	$.438_{(.036)}$
$N_{emb} = 128$	$.850_{(.102)}$	$.417_{(.025)}$	$.427_{(.016)}$
no envelope	$.887_{(.025)}$	$.397_{(.047)}$	$.473_{(.019)}$
$N_{emb} = 128$, no envelope	$.901_{(.030)}$	$.416_{(.014)}$	$.452_{(.008)}$
MAT dist	$.886_{(.024)}$	$.404_{(.045)}$	$.444_{(.012)}$
SchNet dist	$.883_{(.065)}$	$.398_{(.043)}$	$.490_{(.033)}$
$N_{emb} = 128$, SchNet dist	$.888_{(.054)}$	$.400_{(.043)}$	$.445_{(.010)}$

Appendix F: Additional comparison of graph pretraining

F.1 Pretraining methods benchmark

As pretraining is nowadays the main component of big Transformer architectures [4, 6, 36], we decided to devote more attention to this issue. For this purpose, we compared various graph pretraining methods to identify the best one and use it in the final R-MAT model.

Pretraining methods We used various pretraining methods proposed in the molecular property prediction literature [5, 3, 8, 9]. To be more specific, we tried R-MAT with the following pretraining procedures:

- No pretraining – as a baseline we include results for R-MAT fine-tuned from scratch, without any pretraining.
- Masking – masked pretraining used by Maziarka et al. [3]. This is an adaptation of standard MLM pretraining used in NLP [4] to the graphical data. In this approach, we mask features of 15% of all atoms in the molecule and then pass it through the model. The goal is to predict what the masked features were.
- Contextual – contextual pretraining method proposed by Rong et al. [8]. We described it further in the *Pretraining* section.
- Graph-motifs – graph-level motif prediction method proposed by Rong et al. [8], where for every molecule we obtain the fingerprint with information on whether specified molecular functional groups are present in our molecule. The network's task is multi-label classification, where it has to predict, whether every predefined functional group is in the given molecule.
- Physicochemical – graph-level prediction method proposed by Fabian et al. [9]. We described it further in the *Pretraining* section.
- GROVER – pretraining used by authors of GROVER [8]. Combination of two pretraining methods: contextual and graph-motifs.
- R-MAT– pretraining used in this paper. Combination of two pretraining methods: contextual and physicochemical.

Experimental setting We pretrained every model using described earlier dataset with 4M molecules. For every pretraining choice, we trained the model for 50 epochs, using the same training settings as for our standard pretraining (see the *Pretraining* section).

Model hyperparameters and fine-tuning settings were the same as for MAT and GROVER benchmarks. We used the BBBP, ESOL and FreeSolv datasets, split using a scaffold split, with 3 different data splits. The data splits are different than in the previous experiments, and thus the results are different from the results reported in the other paragraphs.

Results Results of this benchmark are presented in Table F.9. We can draw some interesting conclusions from them. One can see, that using any kind of pretraining helps in obtaining better results than for the model trained from scratch. Using physicochemical features for graph-level training gives better results than graph-motifs. Therefore R-MAT pretraining (contextual + physicochemical) is better than GROVER pretraining (contextual + graph-motfis). Moreover combination of two

tasks in pretraining usually gives better results than pretraining using only one task. Interestingly, both node-level pretraining methods (masking and contextual pretraining), return similar results.

Table F.9: Test set performances of R-MAT for different choices of pretraining used.

	BBBP	ESOL	FreeSolv
No pretraining	.855$_{(.081)}$	.423$_{(.021)}$	.495$_{(.016)}$
Masking	.867$_{(.046)}$	.377$_{(.016)}$	.407$_{(.074)}$
Contextual	.901$_{(.039)}$	.382$_{(.034)}$	.413$_{(.047)}$
Graph-motifs	.876$_{(.035)}$	.389$_{(.041)}$	.473$_{(.092)}$
Physiochemical	.897$_{(.042)}$	.406$_{(.072)}$	.400$_{(.085)}$
GROVER	.897$_{(.022)}$	.378$_{(.027)}$	.455$_{(.062)}$
R-MAT	.893$_{(.045)}$	.360$_{(.012)}$	.402$_{(.029)}$

F.2 Pretraining learning curves

In Figure F.4 we present learning curves for R-MAT pretrained with our procedure (contextual + physiochemical). Left-side image shows that training loss flattens out quite quickly; however, it slowly decreases until the end of the training. Moreover one can see that contextual task is harder for the network than graph-level property prediction, as their losses vary by several orders of magnitude. The Left and middle images show that R-MAT predictions for validation datasets are of good quality; moreover, these curves also present that our model learns all the time, reaching the best values at the end of the training.

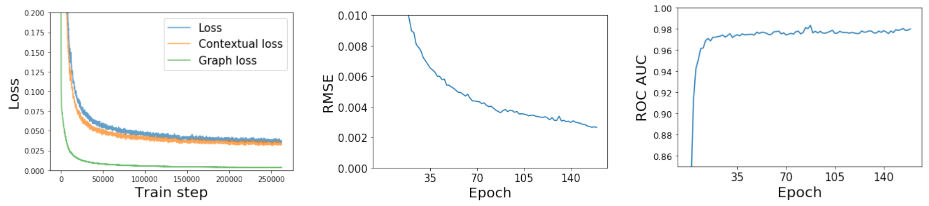

(c) Train loss (d) Validation RMSE for graph-level property prediction task (e) Validation ROC AUC for contextual task

Figure F.4: Learning curves for R-MAT pretraining. On the left-side figure, one can see train losses for both contextual and graph-level property prediction tasks. On the middle figure, one can see the RMSE for graph-level property prediction for the validation dataset obtained by R-MAT during pretraining. On the right-side figure, one can see ROC AUC for the classification task of contextual prediction for validation dataset obtained by R-MAT during pretraining.

We also tested, whether longer pretraining allows R-MAT to get better results during the fine-tuning process. In Figure F.5 we present fine-tuning scores obtained by models pretrained with a different number of pretraining epochs, for FreeSolv and ESOL datasets. Interestingly, longer training does not always results in better fine-tuning scores. This is indeed the case for the FreeSolv dataset (left-side image); however, for ESOL (right-side image) we cannot draw such a conclusion.

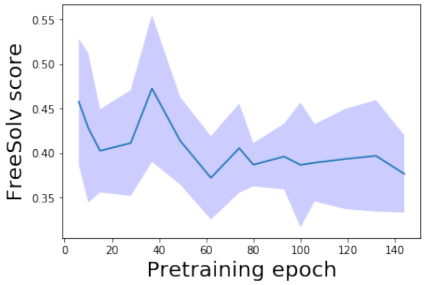

(a) FreeSolv score for R-MAT pretrained for given numbers of pretraining epochs

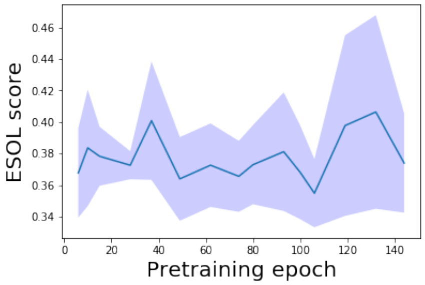

(b) ESOL score for R-MAT pretrained for given numbers of pretraining epochs

Figure F.5: Fine-tuning scores obtained by R-MAT pretrained with a different number of pretraining epochs.

Appendix G: Correlation between graph and 3D distances

We investigate how the distribution of interatomic distances changes when the graph distance between atoms is increased. Figure G.6 shows the interatomic distance distribution for atoms that are n atoms apart. It can be observed, that the variance increases with the graph distance. We conclude that although these distances are correlated, one cannot be easily computed from the other, so including both embeddings can help the model in modeling highly flexible compounds and their stereochemistry.

Appendix H: Running experiments using HuggingMolecules package

In the following section, we present how to run the small hyperparameter budget experiment on the BBBP dataset using the HuggingMolecules package [37]. All the other experiments should be run in the same manner.

1 Download the dataset from `https://osf.io/rgva4/`.
In the directory `data/small_hps_budget/bbbp/` one can find csv file with SMILES and corresponding labels and data splits for this experiment.

2 Run the following command:
```
python -m experiments.scripts.train -d bbbp -m rmat --model.pretrained_name
rmat_4M --train.gpus [1]
```

Author details

References

1. Coley, C.W., Barzilay, R., Green, W.H., Jaakkola, T.S., Jensen, K.F.: Convolutional embedding of attributed molecular graphs for physical property prediction. Journal of chemical information and modeling **57**(8), 1757–1772 (2017)
2. Pocha, A., Danel, T., Podlewska, S., Tabor, J., Maziarka, Ł.: Comparison of atom representations in graph neural networks for molecular property prediction. In: 2021 International Joint Conference on Neural Networks (IJCNN), pp. 1–8 (2021). IEEE
3. Maziarka, Ł., Danel, T., Mucha, S., Rataj, K., Tabor, J., Jastrzębski, S.: Molecule attention transformer. arXiv preprint arXiv:2002.08264 (2020)
4. Devlin, J., Chang, M., Lee, K., Toutanova, K.: BERT: pre-training of deep bidirectional transformers for language understanding. In: Proceedings of the 2019 Conference of the North American Chapter of the Association for Computational Linguistics: Human Language Technologies, NAACL-HLT 2019, Minneapolis, MN, USA, June 2-7, 2019, Volume 1 (Long and Short Papers), pp. 4171–4186 (2019)
5. Hu, W., Liu, B., Gomes, J., Zitnik, M., Liang, P., Pande, V.S., Leskovec, J.: Strategies for pre-training graph neural networks. In: 8th International Conference on Learning Representations, ICLR 2020, Addis Ababa, Ethiopia, April 26-30, 2020 (2020)

6. Liu, Y., Ott, M., Goyal, N., Du, J., Joshi, M., Chen, D., Levy, O., Lewis, M., Zettlemoyer, L., Stoyanov, V.: Roberta: A robustly optimized bert pretraining approach. arXiv preprint arXiv:1907.11692 (2019)
7. Yang, Z., Dai, Z., Yang, Y., Carbonell, J.G., Salakhutdinov, R., Le, Q.V.: Xlnet: Generalized autoregressive pretraining for language understanding. In: Wallach, H.M., Larochelle, H., Beygelzimer, A., d'Alché-Buc, F., Fox, E.B., Garnett, R. (eds.) Advances in Neural Information Processing Systems 32: Annual Conference on Neural Information Processing Systems 2019, NeurIPS 2019, December 8-14, 2019, Vancouver, BC, Canada, pp. 5754–5764 (2019). https://proceedings.neurips.cc/paper/2019/hash/dc6a7e655d7e5840e66733e9ee67cc69-Abstract.html
8. Rong, Y., Bian, Y., Xu, T., Xie, W., Wei, Y., Huang, W., Huang, J.: Self-supervised graph transformer on large-scale molecular data. In: Larochelle, H., Ranzato, M., Hadsell, R., Balcan, M., Lin, H. (eds.) Advances in Neural Information Processing Systems 33: Annual Conference on Neural Information Processing Systems 2020, NeurIPS 2020, December 6-12, 2020, Virtual (2020)
9. Fabian, B., Edlich, T., Gaspar, H., Segler, M., Meyers, J., Fiscato, M., Ahmed, M.: Molecular representation learning with language models and domain-relevant auxiliary tasks. arXiv preprint arXiv:2011.13230 (2020)
10. Landrum, G.: Rdkit: Open-source cheminformatics software (2016)
11. Sterling, T., Irwin, J.J.: Zinc 15–ligand discovery for everyone. Journal of chemical information and modeling **55**(11), 2324–2337 (2015)
12. Gaulton, A., Bellis, L.J., Bento, A.P., Chambers, J., Davies, M., Hersey, A., Light, Y., McGlinchey, S., Michalovich, D., Al-Lazikani, B., Overington, J.P.: ChEMBL: a large-scale bioactivity database for drug discovery. Nucleic Acids Research **40**(D1) (2011)
13. Lipinski, C.A., Lombardo, F., Dominy, B.W., Feeney, P.J.: Experimental and computational approaches to estimate solubility and permeability in drug discovery and development settings. Advanced drug delivery reviews **23**(1-3), 3–25 (1997)
14. Vaswani, A., Shazeer, N., Parmar, N., Uszkoreit, J., Jones, L., Gomez, A.N., Kaiser, L., Polosukhin, I.: Attention is all you need. In: Guyon, I., von Luxburg, U., Bengio, S., Wallach, H.M., Fergus, R., Vishwanathan, S.V.N., Garnett, R. (eds.) Advances in Neural Information Processing Systems 30: Annual Conference on Neural Information Processing Systems 2017, December 4-9, 2017, Long Beach, CA, USA, pp. 5998–6008 (2017)
15. Sergeev, A., Del Balso, M.: Horovod: fast and easy distributed deep learning in tensorflow. arXiv preprint arXiv:1802.05799 (2018)
16. Rogers, D., Hahn, M.: Extended-connectivity fingerprints. Journal of chemical information and modeling **50**(5), 742–754 (2010)
17. Duvenaud, D., Maclaurin, D., Aguilera-Iparraguirre, J., Gómez-Bombarelli, R., Hirzel, T., Aspuru-Guzik, A., Adams, R.P.: Convolutional networks on graphs for learning molecular fingerprints. In: Cortes, C., Lawrence, N.D., Lee, D.D., Sugiyama, M., Garnett, R. (eds.) Advances in Neural Information Processing Systems 28: Annual Conference on Neural Information Processing Systems 2015, December 7-12, 2015, Montreal, Quebec, Canada, pp. 2224–2232 (2015)
18. Yang, K., Swanson, K., Jin, W., Coley, C., Eiden, P., Gao, H., Guzman-Perez, A., Hopper, T., Kelley, B., Mathea, M., *et al.*: Analyzing learned molecular representations for property prediction. Journal of chemical information and modeling **59**(8), 3370–3388 (2019)
19. Kipf, T.N., Welling, M.: Semi-supervised classification with graph convolutional networks. In: International Conference on Learning Representations (2017)
20. Kearnes, S., McCloskey, K., Berndl, M., Pande, V., Riley, P.: Molecular graph convolutions: moving beyond fingerprints. Journal of computer-aided molecular design **30**(8), 595–608 (2016)
21. Gilmer, J., Schoenholz, S.S., Riley, P.F., Vinyals, O., Dahl, G.E.: Neural message passing for quantum chemistry. In: International Conference on Machine Learning, pp. 1263–1272 (2017). PMLR
22. Schütt, K., Kindermans, P.-J., Sauceda Felix, H.E., Chmiela, S., Tkatchenko, A., Müller, K.-R.: SchNet: A continuous-filter convolutional neural network for modeling quantum interactions. In: Advances in Neural Information Processing Systems 30, (2017)
23. Anderson, B., Hy, T.-S., Kondor, R.: Cormorant: Covariant molecular neural networks. arXiv preprint arXiv:1906.04015 (2019)
24. Miller, B.K., Geiger, M., Smidt, T.E., Noé, F.: Relevance of rotationally equivariant convolutions for predicting molecular properties. arXiv preprint arXiv:2008.08461 (2020)
25. Finzi, M., Stanton, S., Izmailov, P., Wilson, A.G.: Generalizing convolutional neural networks for equivariance to lie groups on arbitrary continuous data. In: International Conference on Machine Learning, pp. 3165–3176 (2020). PMLR
26. Thomas, N., Smidt, T., Kearnes, S., Yang, L., Li, L., Kohlhoff, K., Riley, P.: Tensor field networks: Rotation-and translation-equivariant neural networks for 3d point clouds. arXiv preprint arXiv:1802.08219 (2018)
27. Fuchs, F.B., Worrall, D.E., Fischer, V., Welling, M.: Se (3)-transformers: 3d roto-translation equivariant attention networks. arXiv preprint arXiv:2006.10503 (2020)
28. Satorras, V.G., Hoogeboom, E., Welling, M.: E (n) equivariant graph neural networks. arXiv preprint arXiv:2102.09844 (2021)
29. Ramakrishnan, R., Dral, P.O., Rupp, M., Von Lilienfeld, O.A.: Quantum chemistry structures and properties of 134 kilo molecules. Scientific data **1**(1), 1–7 (2014)
30. Shaw, P., Uszkoreit, J., Vaswani, A.: Self-attention with relative position representations. In: Walker, M.A., Ji, H., Stent, A. (eds.) Proceedings of the 2018 Conference of the North American Chapter of the Association for Computational Linguistics: Human Language Technologies, NAACL-HLT, New Orleans, Louisiana, USA, June 1-6, 2018, Volume 2 (Short Papers), pp. 464–468 (2018)
31. Dai, Z., Yang, Z., Yang, Y., Carbonell, J.G., Le, Q.V., Salakhutdinov, R.: Transformer-XL: Attentive language models beyond a fixed-length context. In: Korhonen, A., Traum, D.R., Màrquez, L. (eds.) Proceedings of the 57th Conference of the Association for Computational Linguistics, ACL 2019, Florence,

Italy, July 28- August 2, 2019, Volume 1: Long Papers, pp. 2978–2988 (2019)
32. Huang, Z., Liang, D., Xu, P., Xiang, B.: Improve transformer models with better relative position embeddings. In: Cohn, T., He, Y., Liu, Y. (eds.) Findings of the Association for Computational Linguistics: EMNLP 2020, Online Event, 16-20 November 2020, vol. EMNLP 2020, pp. 3327–3335 (2020)
33. Shang, C., Liu, Q., Chen, K.-S., Sun, J., Lu, J., Yi, J., Bi, J.: Edge attention-based multi-relational graph convolutional networks. arXiv preprint arXiv: 1802.04944 (2018)
34. Brunner, G., Liu, Y., Pascual, D., Richter, O., Ciaramita, M., Wattenhofer, R.: On identifiability in transformers. arXiv preprint arXiv:1908.04211 (2019)
35. Klicpera, J., Groß, J., Günnemann, S.: Directional message passing for molecular graphs. In: 8th International Conference on Learning Representations (2020)
36. Clark, K., Luong, M.-T., Le, Q.V., Manning, C.D.: Electra: Pre-training text encoders as discriminators rather than generators. arXiv preprint arXiv:2003.10555 (2020)
37. Gaiński, P., Maziarka, Ł., Danel, T., Jastrzebski, S.: Huggingmolecules: An open-source library for transformer-based molecular property prediction (student abstract). In: Proceedings of the AAAI Conference on Artificial Intelligence, vol. 36, pp. 12949–12950 (2022)

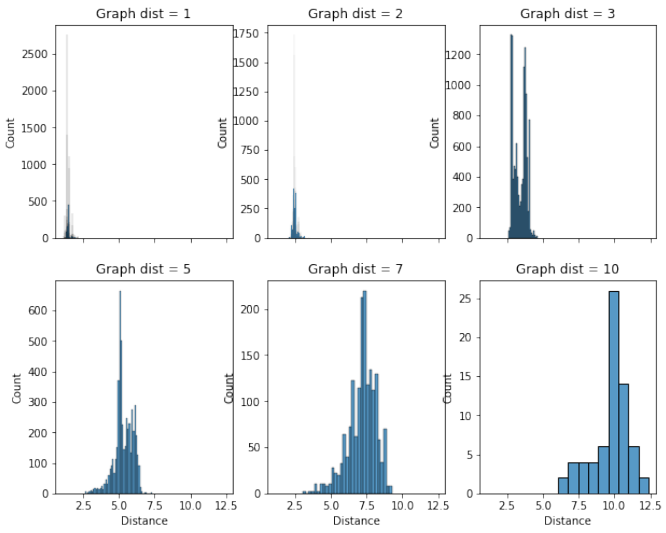

(a) FreeSolv

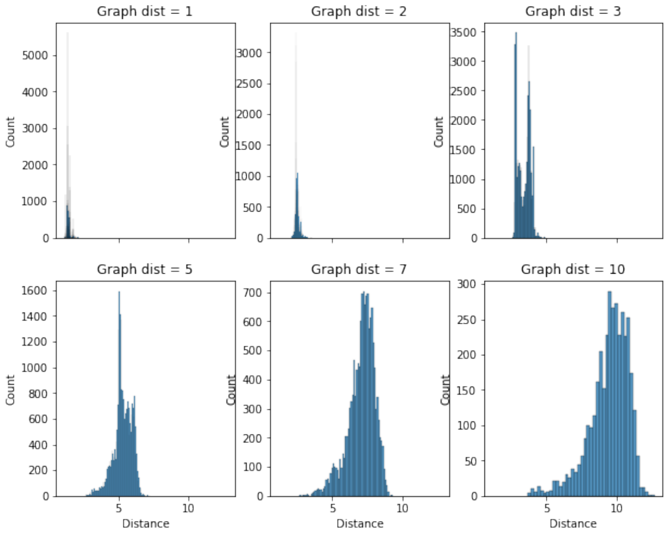

(b) ESOL

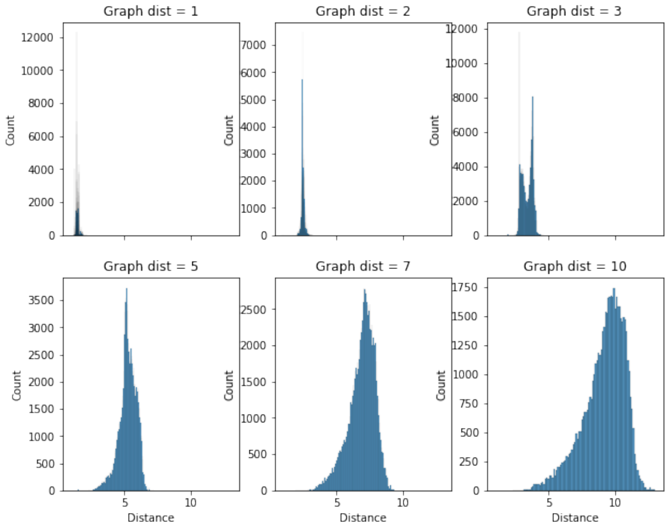

(c) BBBP

Figure G.6: Interatomic distance distribution for atoms that are n atoms apart.
